# Troponin level at presentation as a prognostic factor among patients presenting with non‐ST‐segment elevation myocardial infarction

**DOI:** 10.1002/clc.24166

**Published:** 2023-10-19

**Authors:** Ranel Loutati, Nimrod Perel, Sharon Bruoha, Louay Taha, Meir Tabi, David Marmor, Itshak Amsalem, Rafael Hitter, Mohammed Manassra, Kamal Hamayel, Hani Karameh, Tommer Maller, Yoed Steinmetz, Mohammad Karmi, Mony Shuvy, Michael Glikson, Elad Asher

**Affiliations:** ^1^ Department of Cardiology, Jesselson Integrated Heart Center, Shaare Zedek Medical Center and Faculty of Medicine Hebrew University of Jerusalem Jerusalem Israel; ^2^ Department of Cardiology, Barzilai Medical Center The Ben‐Gurion University of the Negev Beersheba Israel

**Keywords:** NSTEMI, troponin

## Abstract

**Background:**

Timely reperfusion within 120 min is strongly recommended in patients presenting with non‐ST‐segment myocardial infarction (NSTEMI) with very high‐risk features. Evidence regarding the use of high‐sensitivity cardiac troponin (hs‐cTn) concentration upon admission for the risk‐stratification of patients presenting with NSTEMI to expedite percutaneous coronary intervention (PCI) and thus potentially improve outcomes is limited.

**Methods:**

All patients admitted to a tertiary care center ICCU between July 2019 and July 2022 were included. Hs‐cTnI levels on presentaion were recorded, dividing patients into quartiles based on baseline hs‐cTnI. Association between initial hs‐cTnI and all‐cause mortality during up to 3 years of follow‐up was studied.

**Results:**

A total of 544 NSTEMI patients with a median age of 67 were included. Hs‐cTnI levels in each quartile were: (a) ≤122, (b) 123–680, (c) 681–2877, and (d) ≥2878 ng/L. There was no difference between the initial hs‐cTnI level groups regarding age and comorbidities. A higher mortality rate was observed in the highest hs‐cTnI quartile as compared with the lowest hs‐cTnI quartile (16.2% vs. 7.35%, *p* = .03) with hazard ratio (HR) for mortality of 2.6 (95% confidence interval [CI]: 1.23–5.4; *p* = .012) in the unadjusted model, and HR of 2.06 (95% CI: 1.01–4.79; *p* = .047) with adjustment for age, gender, serum creatinine, and significant comorbidities.

**Conclusions:**

Patients with NSTEMI and higher hs‐cTnI levels upon admission faced elevated mortality risk. This underscores the need for further prospective investigations into early reperfusion strategies' impact on NSTEMI patients' mortality, based on admission troponin elevation.

## Graphical abstract

In a study of 544 NSTEMI patients (mean age: 68; 22% females), we explored hs‐cTnI levels on admission as a prognostic marker. The highest hs‐cTnI quartile showed increased mortality (16.2% vs. 7.3%). Adjusted HR for mortality was 2.06 (*p* = .047). Further prospective studies are essential to assess reperfusion strategies based on initial troponin levels.

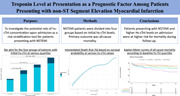

## 1 INTRODUCTION

Although substantial progress has been made in the invasive and pharmacologic therapies of acute coronary syndromes (ACS), the burden of ischemic heart disease is growing with more than 7 million people diagnosed annually with ACS worldwide.^1^ Timely invasive reperfusion is the mainstay of treatment and is associated with a mortality reduction of up to 9% in ST‐segment elevation myocardial infarction (STEMI) and up to 6.5% in non‐ST‐segment elevation myocardial infarction (NSTEMI).^1^, ^2^ However, while immediate revascularization is strongly recommended for patients presenting with STEMI, patients presenting with NSTEMI undergo risk stratification to determine the timing for coronary angiography and reperfusion.^3^, ^4^ Urgent revascularization (<2 h) is recommended in the setting of NSTEMI with very high‐risk features (i.e., hemodynamic instability, recurrent chest pain, malignant arrhythmia, pulmonary congestion related to the MI, and cardiogenic shock). Otherwise, coronary angiography within 24 h is advised. Cardiac biomarkers, in particular high‐sensitivity cardiac troponin (hs‐cTn), have shown promise in risk assessment of ACS patients. Hs‐cTn has predictable kinetics in the setting of acute MI, and peak levels correlate with both ischemic time and quantity of jeopardized myocardium.^5^ Studies have consistently shown that elevated hs‐cTn has high and incremental prognostic value in NSTEMI.^6^, ^7^, ^8^, ^9^, ^10^ In contrast, outcome data is inconsistent when hs‐cTn levels are mildly elevated and/or non‐dynamic.^9^ Current guidelines assign a class I level of recommendation for immediate and serial hs‐cTn measurements for risk and prognostic stratification in NSTEMI,^4^ and urgent reperfusion is recommended in very high‐risk NSTEMI patients.^11^, ^12^ However, these guidelines do not rely on hs‐cTn levels to determine whether the urgent invasive approach is indicated. Hence, the aim of the current study was to evaluate whether high level of hs‐cTn I (hs‐cTnI) on admission correlates with outcomes.

## 2 METHODS

### 2.1 Study population

All patients diagnosed with NSTEMI who were admitted to a tertiary care intensive cardiac care unit (ICCU) at Shaare Zedek Medical Center between July 2019 and July 2022 were prospectively recruited.

The diagnosis of NSTEMI was based on symptoms of myocardial ischemia, new ECG ischemic changes, and a rising and/or falling pattern of high‐sensitivity troponin with at least one value above the 99th percentile URL, according to the ESC guidelines for ACS.^4^

### 2.2 Data collection

Data were anonymously documented in the ICCU by the local coordinator and prospectively submitted into an electronic case report form. Data were checked for accuracy and out‐of‐range values by the coordinating unit. Demographic data, presenting symptoms, comorbid conditions, and physical examination were systematically recorded. Laboratory, imaging, angiographic results, and clinical course data were collected as well.

Patients were followed for up to 3 years after presentation. Patients were divided into four quartiles according to their hs‐cTnI level on admission. The first quartile served as the reference group. Hs‐cTnI assay was determined in a central laboratory (ARCHITECT STAT hs‐cTnI immunoassay) with a 99th percentile reference level of 17 ng/L for females and 35 ng/L for males.^13^ Invasive and pharmacologic treatments were in accordance with the European Society of Cardiology guidelines for ACS.^13^

The Institutional Review Board approved the study based on strict maintenance of participants' anonymity by de‐identifying during database analysis. No individual consent was obtained. Moreover, the authors have no conflicts of interest to declare. No funding was applied to the study. All methods were performed in accordance with the relevant guidelines and regulations.

### 2.3 Study outcomes

The primary outcome was all‐cause mortality with a follow‐up time of up to 3 years. The overall mortality rate was determined from the Israeli Ministry of Internal Affairs.

### 2.4 Statistical analysis

Continuous variables were expressed as mean ± standard deviation if normally distributed or median with interquartile range if skewed. Categorical variables were presented as frequency (%). Continuous data were compared with the Student's *t* test and categorical data were compared with the use of the chi‐square test or Fisher exact test where appropriate. Differences between the four initial hs‐cTnI groups were analyzed using one‐way ANOVA for continuous variables that were normally distributed, while the Kruskal–Wallis test was used to compare continuous variables that did not adhere to a normal distribution. Multiple comparisons for continuous and categorical variables were tested using Bonferroni's correction. For survival analysis, patients were censored in the case of death or diagnosis of malignant cancer during follow‐up. The probability of death according to the study groups was graphically displayed according to the method of Kaplan–Meier, with a comparison of cumulative survival across strata by the log‐rank test. Univariate and multivariate Cox proportional hazards regression modeling was used to compare patients presenting with hs‐cTnI levels in the fourth quartile to patients with initial hs‐cTnI levels in the first quartile, with adjustments made for parameters that were found to be significant in the univariate model or are recognized as potential confounders of hs‐cTnI concentrations. The adjusted Cox model incorporated the following variables: age, gender, serum creatinine, and comorbidities including arterial hypertension, diabetes mellitus (DM), obesity (BMI ≥ 30), previous myocardial infarction (MI), and clinical diagnosis of congestive heart failure (CHF). All analyses were performed using R software version 3.4.4 (R Foundation for Statistical Computing). An association was considered statistically significant for a two‐sided *p* value of less than .05.

## 3 RESULTS

### 3.1 Baseline characteristics

A total of 665 patients with NSTEMI were screened; of them, 27 were excluded due to missing data, and 94 were excluded due to loss of follow‐up. Hence, the final study population included 544 patients. The mean age was 68 (±11) years old and 122 (22%) were female. Patients' characteristics are present in Table 1. Three hundred and fifty (64%) patients suffered from hypertension, 233 (43%) from DM, 342 (63%) from dyslipidemia, 184 (34%) patients were active smokers, and 39 (7%) patients had previous MI.

**Table 1:** Baseline characteristics.

Clinical Variables	Initial hs‐cTnI < 122 ng/L (25th percentile) (*n* = 136) (25%)	Initial hs‐cTnI between 122 and 680 ng/L (50th percentile) (*n* = 136) (25%)	Initial hs‐cTnI between 680 and 2878 ng/L (75th percentile) (*n* = 136) (25%)	Initial hs‐cTnI ≥ 2878 ng/L (75th percentile) (*n* = 136) (25%)	Overall (*n* = 544)	*p* Value
Age in years (median [IQR])	66 (58–75)	65 (58–78)	67 (59–76)	69 (60–79)	67 (58–77)	.803
Female sex—no. (%)	22 (16.2%)	41 (30.1%)	36 (26.5%)	23 (16.9%)	122 (22.4%)	**.0227**
BMI mean (SD)	28 (4.49)	28 (5.16)	28.5 (5.15)	28.3 (5)	28.2 (4.95)	.909
Hypertension—no. (%)	93 (68.4%)	93 (68.4%)	80 (58.8%)	84 (61.8%)	350 (64.3%)	.388
DM—no. (%)	53 (39%)	65 (47.8%)	56 (41.2%)	59 (43.4%)	233 (42.8%)	.669
Hyperlipidemia—no. (%)	94 (69.1%)	89 (65.4%)	82 (60.3%)	77 (56.6%)	342 (62.9%)	.256
Smoking—no. (%)	38 (27.9%)	47 (34.6%)	46 (33.8%)	53 (39%)	184 (33.8%)	.442
Prior CAD—no. (%)	63 (46.3%)	60 (44.1%)	47 (34.6%)	46 (33.8%)	216 (39.7%)	.133
Prior CABG—no. (%)	10 (7.43%)	13 (9.6%)	7 (5.1%)	12 (8.8%)	42 (7.7%)	.705
CVA—no. (%)	7 (5.1%)	10 (7.4%)	12 (8.8%)	12 (8.8%)	41 (7.5%)	.778
PAD—no. (%)	4 (2.9%)	5 (3.7%)	11 (8.1%)	12 (8.8%)	32 (5.9%)	.156
CHF or CMP—no. (%)	8 (5.9%)	11 (8.1%)	6 (4.4%)	17 (12.5%)	42 (7.7%)	.13
COPD—no. (%)	6 (4.4%)	13 (9.6%)	9 (6.6%)	7 (5.1%)	35 (6.4%)	.476
Atrial fibrillation—no. (%)	12 (8.8%)	10 (7.4%)	14 (10.3%)	9 (6.6%)	45 (8.3%)	.839
CKD—no. (%)	15 (11%)	17 (12.5%)	8 (5.9%)	21 (15.4%)	61 (11.2%)	.161
S/P Malignancy—no. (%)	5 (3.7%)	9 (6.6%)	12 (8.8%)	14 (10.3%)	40 (7.4%)	.291
EF (%), mean (SD)	49.5 (11)	49.1 (10.5)	48.7 (10.7)	48.3 (11.1)	48.9 (10.8)	.917
Serum creatinine, mg/dL, mean (SD)	1.1 (0.2)	1.25 (0.6)	1.2 (0.4)	1.28 (0.4)	1.23 (0.4)	.586
Albumin, g/dL, mean (SD)	3.45 (0.536)	3.88 (0.464)	3.73 (0.38)	3.72 (1.217)	3.75 (0.435)	.593
Peak hs‐cTnI, ng/L, mean (SD)	10 000 (23 200)	9690 (27 100)	9430 (19 400)	36 100 (63 100)	16 300 (39 100)	**<.001**
HDL, mg/dL, mean (SD)	41.1 (11.5)	39.8 (12.3)	40.3 (11.6)	39 (12.1)	40.1 (11.9)	.718
LDL, mg/dL, mean (SD)	97.6 (40.8)	92.6 (38)	99 (39.6)	99.2 (38.8)	97.6 (40)	.535
CRP, mg/L, mean (SD)	0.753 (1.21)	1.43 (1.3)	2.2 (1.7)	3.6 (2.1)	2 (2.3)	**<.001**
HbA1c (%), mean (SD)	6.41 (1.38)	6.35 (1.32)	6.46 (1.63)	6.53 (1.63)	6.44 (1.49)	.926
TSH, mIU/mL, mean (SD)	1.77 (1.33)	1.99 (1.56)	2.12 (1.47)	1.92 (1.55)	1.95 (1.48)	.426
IPF (%), mean (SD)	5.09 (2.81)	5.55 (2.38)	5.16 (2.97)	5.35 (2.86)	5.28 (2.76)	.77
Culprit vessel—no. (%)					254 (46.7%)	
LAD	70 (51.5%)	58 (42.6%)	60 (44.1%)	66 (48.6%)	107 (19.7%)	.452
RCA	22 (16.1%)	29 (21.3%)	27 (19.8%)	29 (21.3%)	96 (17.6%)	.728
Cx	20 (14.7%)	24 (17.7%)	25 (18.4%)	27 (19.8%)	87 (16%)	.138
Other/unknown	24 (17.7%)	25 (18.4%)	24 (17.7%)	14 (10.3%)		

Abbreviations: BMI, body mass index; CABG, coronary artery bypass graft surgery; CAD, coronary artery disease; CKD, chronic kidney disease; CHF, congestive heart failure; CMP, cardiomyopathy; COPD, chronic obstructive pulmonary disease; CRP, C‐reactive protein; CVA, cerebrovascular accident; Cx, circumflex artery; DM, diabetes mellitus; EF, ejection fraction; HbA1c, hemoglobin A1C; HDL, high‐density lipoprotein; hs‐cTnI, high sensitivity cardiac troponin I; IPF, immature platelet fraction; LAD, left anterior descending; LDL, low‐density lipoprotein; PAD, peripheral artery disease; RCA, right coronary artery; TSH, thyroid stimulating hormone.

### 3.2 Hs‐cTnI levels at presentation

Hs‐cTnI levels in each quartile were: (a) ≤122, (b) 123–680, (c) 681–2877, and (d) ≥2878 ng/L. The median initial hs‐cTnI level was 680 ng/L (range 0–150 008 ng/L). There were no differences regarding age or comorbidities between the four initial hs‐cTnI level quartiles. Prevalence of male gender, higher peak level of hs‐cTnI during admission, and C‐reactive protein (CRP) were greater in the fourth quartile than in the first quartile (Table 1). Stratifying NSTEMI patients based on gender resulted in distinct cutpoints. In male patients, the cutpoints were as follows: (a) ≤217, (b) 218–698L, (c) 699–3276, and (d) ≥3277 ng/L. Conversely, female patients exhibited lower values, as anticipated, with the following cutpoints: (a) ≤100, (b) 101–612, (c) 613–2449, and (d) ≥2450 ng/L.

### 3.3 Interventions and complications during admission

Specific procedures performed during the ICCU admission course are reported in Supporting Information: Table S1. PCI was performed in 369 (68%) patients, coronary angiography without intervention was performed in 119 (22%), coronary artery bypass grafting (CABG) was performed in 27 (4.9%) patients, and medication therapy alone was assigned to only 29 (5.1%) of the patients. There were no differences in the treatments between the patients in all quartiles, as shown in Supporting Information: Table S1. The overall complication rate during admission was 15% with no significant differences between the quartiles, as presented in Supporting Information: Table S2. Acute renal failure (ARF) and shock were more common in the fourth quartile than in the first quartile (7.4% vs. 2.9%, *p* = .07% and 9.5% vs. 5.1%, *p* = .062, respectively).

### 3.4 Mortality rate during follow‐up

**Figure 1:**
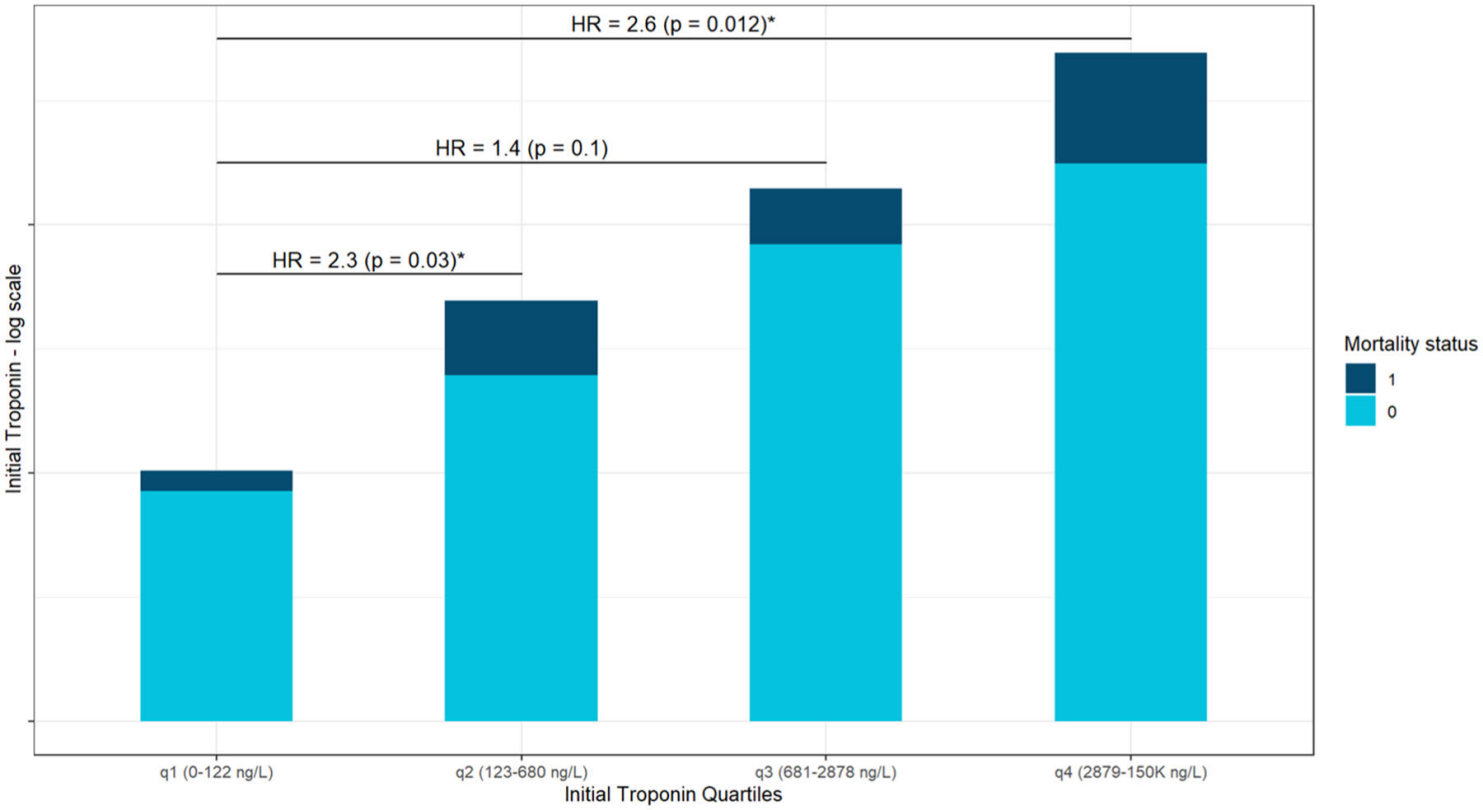
This bar plot shows the relative portion of deceased patients in each of the four initial hs‐cTnI groups of patients. Hazard ratios (HRs) for mortality between the first quartile and the three others are also depicted, indicating greater risk of mortality as initial hs‐cTnI rises. hs‐cTnI, high‐sensitivity cardiac troponin I.

During the follow‐up period death rate was 10 (7.3%), 24 (17.6%), 14 (10.3%), and 22 (16.2%) for each quartile, respectively, as shown in Figure 1. Age, left ventricular ejection fraction, previous MI, CHF, hypertension, and DM were all predictors of poor survival in a univariate Cox model (Table 2).

**Table 2:** Univariate Cox model.

Clinical variables	Hazard ratio	95% CI	*p* Value
Age [years] (continuous)	1.05	1.02–1.07	<.001
Sex (male)	0.65	0.39–1.08	.1
BMI [kg/m^2^] (continuous)	0.99	0.94–1.04	.7
Obesity (BMI > 30)	0.9	0.54–1.50	.7
LVEF [%] (continuous)	0.94	0.91–0.97	<.001
Low‐LVEF (<41%)	2.87	1.75–4.70	<.001
PCI	0.48	0.29–0.81	<.001
previous MI	2.90	1.56–5.40	<.001
Heart failure (clinical diagnosis)	2.78	1.49–5.20	.001
Arterial hypertension	2.71	1.42–5.17	.002
Diabetes mellitus	1.68	1.04–2.70	.032
COPD	1.40	0.66–2.94	.4
Dyslipidemia	1.30	0.79–2.14	.3
Cerebrovascular accident	1.33	0.61–2.91	.5
Peripheral vascular disease	2.41	1.23–4.71	.01
Smoking	0.46	0.26–0.83	.01
Malignancy	1.23	0.56–2.68	.6

Abbreviations: BMI, body mass index; CI, confidence interval; COPD, chronic obstructive pulmonary disease; LVEF, left ventricle ejection fraction; MI, myocardial infarction; PCI, percutaneous coronary intervention.

**Figure 2:**
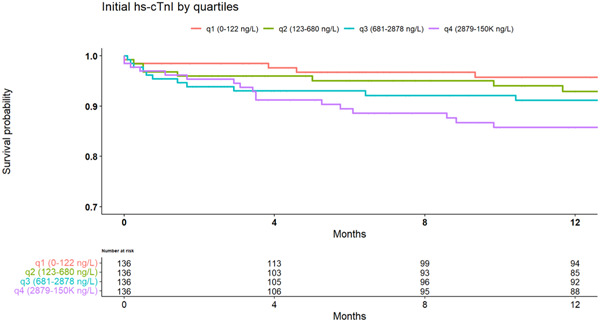
Kaplan–Meier survival curves during the median follow‐up period demonstrate lower survival probability in patients with higher initial hs‐cTnI levels. Log‐rank *p* = .026. hs‐cTnI, high‐sensitivity cardiac troponin I.

The cumulative probability of death at median follow‐up time (1 year) was 4.2 ± 1.8%, 7 ± 2.4%, 8.8 ± 2.5%, and 14.2 ± 3.2% for each quartile, respectively (Figure 2; log‐rank *p* = .026 for overall difference during follow‐up). Patients who presented with initial hs‐cTnI in the fourth quartile were 2.6 times more likely to die at 1‐year follow‐up, as compared with patients in the first quartile (HR: 2.6; 95% CI: 1.23–5.4, *p* = .012). A multivariate Cox model adjusted for age, gender, serum creatinine, and comorbidities including arterial hypertension, diabetes mellitus, obesity (BMI ≥ 30), previous MI, and CHF showed consistent results (HR: 2.06; 95% CI: 1.01–4.79, *p* = .047) as was further adjustment of the model for revascularization (PCI and CABG) during admission (HR: 1.96; 95% CI: 0.94–4.31, *p* = .06).

### 3.5 Dose–response effect of initial hs‐cTnI levels on mortality

**Figure 3:**
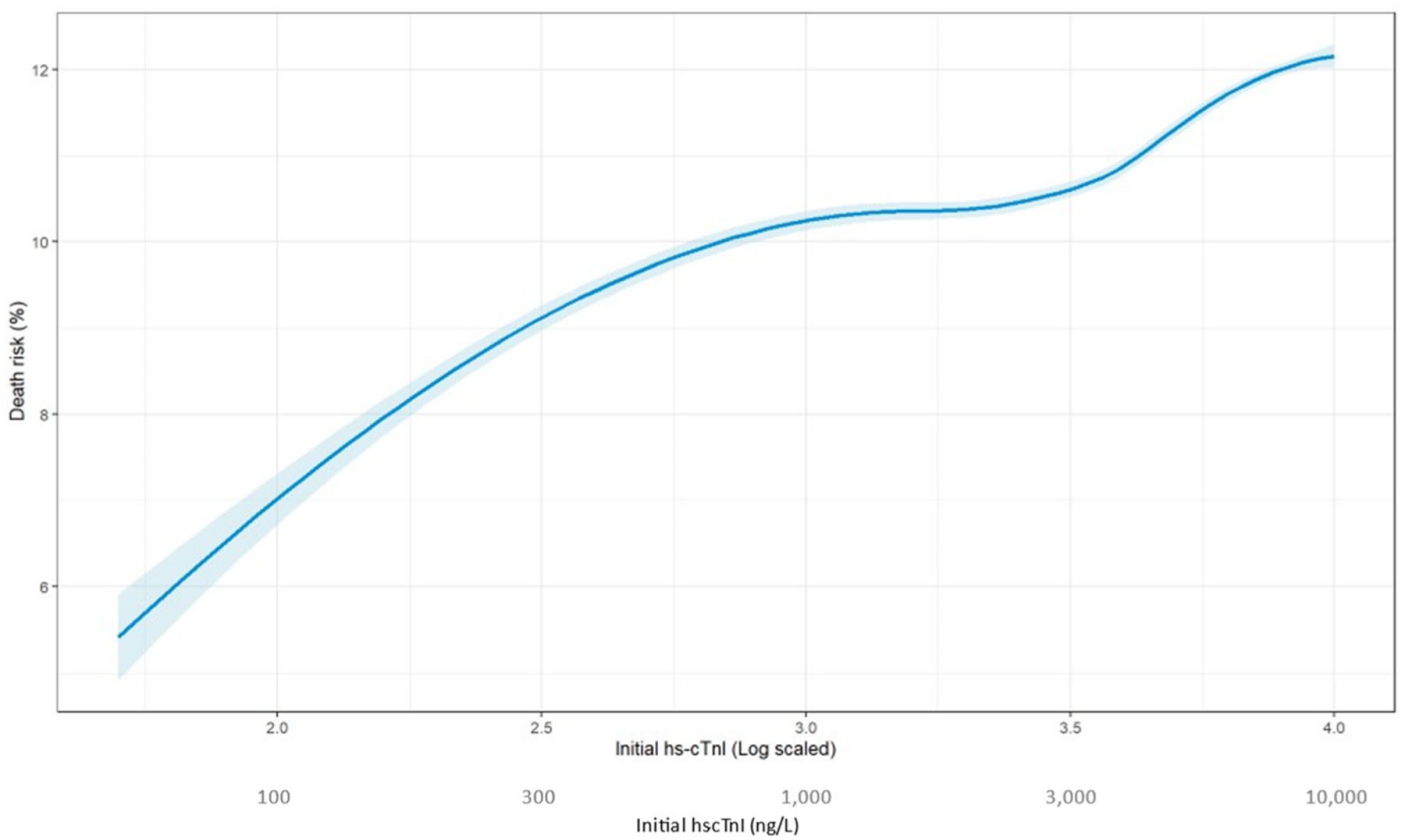
This graph represented the interpolated risk for mortality at 1 year of presentation, based on initial hs‐cTnI levels. As initial hs‐cTnI levels increased, so is the risk for mortality. A second, later peak, in the death risk is seen when initial hs‐cTnI levels reach the fourth quartile. hs‐cTnI, high‐sensitivity cardiac troponin I.

To investigate the potential dose–response relationship between initial hs‐cTnI and mortality, we plotted the risk of death at 1 year of follow‐up using Kaplan–Meier survival analysis across various levels of initial hs‐cTnI, as depicted in Figure 3. The plot exhibits a sigmoidal curve, characterized by a gradual increase in mortality with rising levels of initial hs‐cTnI, until reaching a plateau at hs‐cTnI = 1000 ng/L. Notably, a second peak in mortality risk emerges at hs‐cTnI = 2878 ng/L.

## 4 DISCUSSION

The main findings of our study are (a) we have demonstrated that in patients presenting with NSTEMI, elevated baseline hs‐cTnI levels were associated with increased risk of 3‐year all‐cause mortality, (b) there was a dose–response relationship between baseline hs‐CTnI and mortality with highest risk of mortality observed in patients with highest baseline hs‐cTnI levels, and (c) even after adjusting the baseline hs‐cTnI mortality association for potential confounders, initial high hs‐cTnI levels remained an independent predictor of all‐cause mortality.

To the best of our knowledge, this study is the first to examine the association between hs‐cTnI levels upon hospital admission and long‐term overall survival in patients diagnosed with NSTEMI. Prior studies that have examined the association between initial troponin levels and mortality have included patients with a broader range of diagnoses, encompassing all types of acute myocardial infraction^14^ or patients presenting with chest pain.^15^ Another significant distinction between our study and previous ones is our focus on examining troponin levels at presentation, as opposed to peak troponin levels or changes in troponin during admission course, which has been linked to adverse clinical outcomes and mortality.^15^, ^16^, ^17^

Our first finding further supports the conclusions of Antman et al. regarding the role of troponin level upon hospital admission and short‐term mortality in ACS patients.^18^ The identification of a higher‐risk subgroup with hs‐cTnI levels greater or equal to 2878 ng/L is very intriguing, as this subgroup did not exhibit any distinctive baseline characteristics or complications. We hypothesized that the heightened risk of mortality in those patients is likely a result of later changes that occur in the myocardium of NSTEMI patients, such as remodeling, which is related to elevated risk of death.^19^ Hence, there were no between‐group differences in the short‐term period. In a large observational study comprising 4123 patients with non‐ST‐segment elevation ACS, Kontos et al. demonstrated a gradual rise in cardiac mortality as peak cTnI levels increased at 30‐day and 6‐month follow‐up intervals.^15^ Our results are consistent with these findings. However, our aim was to investigate specifically the association between initial cTnI levels and mortality over a longer follow‐up period. From a clinical perspective, our results might have greater clinical implications due to the dynamic nature of ACS patients.

Bagai et al. showed that there is a differential relationship between the magnitude of peak troponin elevation and long‐term mortality in ACS patients treated with and without revascularization.^16^ Our findings also suggest better prognosis after revascularization regarding the association between initial hs‐cTnI levels and mortality, although this was not the aim of our study and it was not powered for that. The association between initial hs‐cTnI levels and mortality, and the lower mortality rate in revascularized patients, imply that initial hs‐cTnI levels may be useful in determining the need for urgent revascularization in NSTEMI patients. Current NSTEMI guidelines advise revascularization within 120 min in patients with very high‐risk characteristics,^4^ regardless of the level of cardiac biomarkers. However, based on our results and previous important studies, initial hs‐cTnI may further contribute to the decision‐making process for urgent revascularization among NSTEMI patients, to reduce the higher mortality rates that we have observed in NSTEMI patients with high baseline hs‐cTnI levels.

### 4.1 Study limitations

Our study has several limitations: (1) The study was conducted in a single tertiary‐care ICCU, resulting in a relatively small study population of 544 patients. Nevertheless, we included all‐comers in a real‐life setting. (2) Our analysis was based on overall mortality rather than cardiovascular mortality. Though mortality statistics in Israel closely resemble those of the European Union, where cardiovascular death is the second most prevalent cause of death following cancer.^20^ (3) There may be unmeasured clinical variables that could explain our observations, including the time elapsed between symptom onset to hs‐cTnI testing, details regarding coronary angiography beyond the culprit vessel, including lesion description and the exact type of stents employed in the PCI group. (4) While independent associations have been demonstrated, causality could not be established due to study design; hence, the superiority of one treatment modality over another is not supported by our findings.

## 5 CONCLUSION

Despite the prognostic implications of troponin levels in ACS, current guidelines for urgent revascularization in NSTEMI patients do not incorporate this biomarker in their decision‐making process regarding treatment. In our study, we have demonstrated an association between hs‐cTnI levels upon admission and overall survival in patients diagnosed with NSTEMI. Furthermore, we identified a subgroup that is at a heightened risk of mortality according to their initial hs‐cTnI level. Interestingly, this association becomes less significant when early reperfusion strategy is considered. These findings may suggest that initial hs‐cTnI levels could be an additional factor when determining reperfusion strategies in NSTEMI patients. Further prospective studies are warranted to investigate the impact of early reperfusion strategies on mortality in NSTEMI patients categorized by their initial presenting troponin levels.

## CONFLICT OF INTEREST STATEMENT

The authors declare no conflict of interest.

## Supporting information

Supporting information.

## Data Availability

Data were generated at Shaare Zedek Medical Center. Derived data supporting the findings of this study are available from the corresponding author (R. L.) upon request.
