# Supplementary material for: Troponin level at presentation as a prognostic factor among patients presenting with non‐ST‐segment elevation myocardial infarction
Source: Clin Cardiol. 2023 Oct 19;47(1):e24166. doi: 10.1002/clc.24166 (PMC10766125; doi:10.1002/clc.24166)
Supplement: Supplementary file 1 — Supporting information. [file CLC-47-e24166-s001.docx]

Supplementary Tables for Manuscript:

Troponin Level at Presentation as a Prognostic Factor Among Patients Presenting with non-ST Segment Elevation Myocardial Infarction

Supplementary Table S1 – Interventions during admission

| **Type of procedure** | **Initial hs-cTnI < 122 ng/L (25^th^ percentile)**  **(n=136) (25%)** | **Initial hs-cTnI between 122 ng/L – 680 ng/L (50^th^ percentile)**  **(n=136) (25%)** | **Initial hs-cTnI between 680 ng/L – 2878 ng/L (75^th^ percentile)**  **(n=136) (25%)** | **Initial hs-cTnI ≥ 2878 ng/L (75^th^ percentile) (n=136) (25%)** | **Overall (n=544)** | **P-Value** |
| --- | --- | --- | --- | --- | --- | --- |
| **Urgent PCI (< 2hr) – no. (%)** | 17 (12.5%) | 9 (6.6%) | 16 (11.8%) | 8 (5.9%) | 50 (9.2%) | 0.221 |
| **PCI – no. (%)** | 72 (52.9%) | 86 (63.2%) | 80 (58.8%) | 81 (59.6%) | 319 (58.6%) | 0.549 |
| **Coronary angiography– no. (%)** | 31 (22.8%) | 29 (21.3%) | 31 (22.8%) | 28 (20.6%) | 119 (21.9%) | 0.99 |
| **CABG – no. (%)** | 8 (5.9%) | 4 (3%) | 8 (5.9%) | 7 (5.1%) | 27 (5%) | 0.681 |
| **Conservative therapy – no. (%)** | 8 (5.9%) | 8 (5.9%) | 1 (0.7%) | 12 (8.8%) | 29 (5.1%) | 0.669 |
| **Pacemaker/ICD – no. (%)** | 3 (2.2%) | 0 (0%) | 2 (1.5%) | 1 (0.7%) | 6 (1.1%) | 0.498 |
| **CPR – no. (%)** | 4 (2.9%) | 1 (0.7%) | 2 (1.5%) | 2 (1.5%) | 9 (1.7%) | 0.709 |
| **Mechanical Ventilation – no. (%)** | 8 (5.9%) | 5 (3.7%) | 4 (2.9%) | 5 (3.7%) | 22 (4%) | 0.79 |
| **RRT – no. (%)** | 4 (3%) | 9 (6.6%) | 7 (5.1%) | 7 (5.1%) | 27 (5%) | 0.432 |
| **IABP – no. (%)** | 2 (1.5%) | 2 (1.5%) | 2 (1.5%) | 5 (3.7%) | 11 (2%) | 0.654 |

PCI=Percutaneous Coronary Intervention; Cath.=Coronary angiography without intervention; CABG=Coronary Artery Bypass Graft; ICD=Implantable Cardioverter Defibrilator; CPR=Cardio-Pulmonary Resuscitation; RRT=Renal replacement therapy; IABP=Intra-Aortic baloon pump

Supplementary Table S2 – Complications during admission

| **Type of complication** | **Initial hs-cTnI < 122 ng/L (25^th^ percentile)**  **(n=136) (25%)** | **Initial hs-cTnI between 122 ng/L – 680 ng/L (50^th^ percentile)**  **(n=136) (25%)** | **Initial hs-cTnI between 680 ng/L – 2878 ng/L (75^th^ percentile)**  **(n=136) (25%)** | **Initial hs-cTnI ≥ 2878 ng/L (75^th^ percentile) (n=136) (25%)** | **Overall (n=544)** | **P-Value** |
| --- | --- | --- | --- | --- | --- | --- |
| **Malignant Arrhythmia – no. (%)** | 2 (1.5%) | 0 (0%) | 1 (0.7%) | 2 (1.5%) | 5 (0.9%) | 0.695 |
| **Shock* – no. (%)** | 7 (5.1%) | 6 (4.4%) | 6 (4.4%) | 13 (9.5%) | 32 (5.9%) | 0.062 |
| **Stroke/TIA. – no. (%)** | 0 (0%) | 1 (0.7%) | 3 (2.2%) | 1 (0.7%) | 5 (0.9%) | 0.695 |
| **ARF – no. (%)** | 4 (2.9%) | 2 (1.5%) | 3 (2.2%) | 10 (7.4%) | 19 (3.5%) | 0.07 |
| **Significant bleed – no. (%)** | 0 (0%) | 1 (0.7%) | 3 (2.2%) | 5 (3.7%) | 9 (1.7%) | 0.155 |

ARF=Acute Renal Failure

*any type of shock
